# Metagenomic Sequencing Analysis of the Effects of Colistin Sulfate on the Pig Gut Microbiome

**DOI:** 10.3389/fvets.2021.663820

**Published:** 2021-07-02

**Authors:** Ling Guo, Dan Zhang, Shulin Fu, Jiacheng Zhang, Xiaofang Zhang, Jing He, Chun Peng, Yunfei Zhang, Yinsheng Qiu, Chun Ye, Yu Liu, Zhongyuan Wu, Chien-An Andy Hu

**Affiliations:** ^1^Hubei Key Laboratory of Animal Nutrition and Feed Science, Wuhan Polytechnic University, Wuhan, China; ^2^Hubei Collaborative Innovation Center for Animal Nutrition and Feed Safety, Wuhan, China; ^3^Biochemistry and Molecular Biology, University of New Mexico School of Medicine, Albuquerque, NM, United States

**Keywords:** colistin sulfate, microbiome, metagenomic sequencing, pig gut, antibiotic resistance

## Abstract

The gut microbiome plays important roles in maintaining host health, and inappropriate use of antibiotics can cause imbalance, which may contribute to serious disease. However, despite its promise, using metagenomic sequencing to explore the effects of colistin on gut microbiome composition in pig has not been reported. Herein, we evaluated the roles of colistin in gut microbiome modulation in pigs. Metagenomic analysis demonstrated that overall microbial diversity was higher in the colistin group compared with the control group. Antibiotic Resistance Genes Database analysis demonstrated that following colistin treatment, expression levels of *tsnr, ant6ia, tetq, oleb, norm, ant3ia*, and *mexh* were significantly upregulated, indicating that colistin may induce transformation of antibiotic resistance genes. Colistin also affected the microbiome distribution patterns at both genus and phylum levels. In addition, at the species level, colistin significantly reduced the abundance of *Prevotella copri, Phascolarctobacterium succinatutens*, and *Prevotella stercorea* and enhanced the abundance of *Treponema succinifaciens* and *Acidaminococcus fermentans* compared to the control group. Gene Ontology analysis demonstrated that following treatment with colistin, metabolic process, cellular process, and single-organism process were the dominant affected terms. Kyoto Encyclopedia of Genes and Genomes analysis showed that oxidative phosphorylation, protein processing in endoplasmic reticulum, various types of N-glycan biosynthesis, protein processing in endoplasmic reticulum, pathogenic *Escherichia coli* infection, and mitogen-activated protein kinase signaling pathway–yeast were the dominant signaling pathways in the colistin group. Overall, our results suggested that colistin affects microbial diversity and may modulate gut microbiome composition in pig, potentially providing novel strategy or antibiotic rationalization pertinent to human and animal health.

## Introduction

Microbes play important roles in maintaining host health, and research is increasingly showing that the intestinal microbiome is involved in crosstalk between the gut and the host immunocompetence state (1). Imbalance in gut microbiota function may lead to serious diseases (2). Changes in the human gut microbiota have been linked to neurodegenerative disorders such as Alzheimer disease (3). The gut microbiota plays an important role in fiber metabolism in pigs (4). Dysbiosis of the gut microbiota can cause intestinal immune dysfunction in piglets (5). The gut microbiota is involved in the gut inflammation and lipid metabolism in weaned piglets (6). Environmental changes may contribute to the alteration of microbiomes, such as overuse of antibiotics (7). Therefore, it is beneficial to study the relationships between antibiotics and gut microbiota to develop potential strategies for maintaining health and preventing or fighting disease.

Colistin is a key antibiotic used to treat multidrug-resistant Gram-negative bacterial infections (8). Previous research has shown that colistin can effectively cure osteomyelitis induced by carbapenemase-producing *Klebsiella pneumoniae* (9). Colistin is used to treat urinary tract infections triggered by extremely drug-resistant *Pseudomonas aeruginosa* (10). The efficiency of the treatment of bacterial infection can vary, and side effects of colistin have been reported, such as peripheral neurotoxicity and mitochondrial dysfunction (11). Also, extensive use and abuse of colistin have resulted in drug resistance through selection pressure. A mobile colistin resistance (mcr) gene (mcr-10) has been discovered in *Enterobacter roggenkampii* (12), and mcr-1 was identified in *Escherichia coli* (13). Furthermore, some colistin-resistant bacterial and mcr family genes can be transmitted through the food chain (14). Thus, using colistin as an additive to maintain and enhance animal growth has been banned (15). Previous studies showed that colistin can affect fecal microbiome composition in mice (16). Using 16S rRNA sequencing, colistin was found to alter the piglet intestinal microbiota (17, 18). However, so far, using metagenomic sequencing analysis to explore the effects of colistin on the pig gut microbiome composition has not been reported.

Modification of the gut microbiome in pig can be triggered by feed additives supplemented with antibiotics (19–21). However, the methods used in these studies to analyze the overall microbiome community composition were based on 16S RNA sequencing or polymerase chain reaction. Disadvantages of these technologies include an inability to fully determine the composition of the microbiome and the abundance of microbiota genes and to identify diagnostic markers, which limits applications in microbiota analysis (22). Thus, it is important to adopt a high-quality and high-throughput sequencing approach for gut microbial community function analysis in pig and other animals. Metagenomics sequencing analysis is a credible tool that can investigate in detail the functions of the gut microbiome in pig (23).

The main objective of the present study was to investigate the effects of colistin on gut microbiome composition changes in pig. Our results showed that colistin could modulate the pig gut microbiome composition. The findings provide guidance for rational antibiotic use in the clinic, and a novel strategy for maintaining gut health in humans and other animals.

## Materials and Methods

This study was carried out in strict accordance with the recommendations of the China Regulations for the Administration of Affairs Concerning Experimental Animals 1988 and the Hubei Regulations for the Administration of Affairs Concerning Experimental Animals 2005. The protocol was approved by the China Hubei Province Science and Technology Department (Permit No. SYXK[ER] 2010-0029). All animal experiments and animal care procedures were approved by the Animal Care and Use Committee of Wuhan Polytechnic University, Hubei Province, China (EM950, November 5, 2020). All experimental animals were euthanized at the end of the experiment.

### Drugs and Animals

Colistin sulfate was obtained from Livzon Group Fuzhou Fuxing Pharmaceutical Co. LTD (Fuzhou, China). Six 30-day-old naturally farrowed early-weaned piglets (Duroc × Landrace × large white), weighing 8 to 10 kg, were purchased from Wuhan Wannianqing Animal Husbandry Co. Ltd. (Wuhan, China) for *in vivo* experiments.

### Experiment Design

The six piglets were administered with basic diet for 7 days, and stools were collected as the control group. Piglets were then fed basic diet supplemented with 20 g colistin sulfate/t. After 14 days, stools from piglets were collected as the colistin group. Samples from control and colistin groups were used to metagenomic sequencing.

### Isolation of Genomic DNA From Pig Stools

Stool samples were collected, immediately snap-frozen under liquid nitrogen, and stored at −80°C until use. Genomic DNA from stools was isolated using a Qiagen Qiamp Fast DNA Stool Mini Kit (Qiagen, Germany) according to the manufacturer's instructions. The quality of the isolated DNA was assessed by a NanoDrop instrument (Thermo Scientific, USA) and genomic DNA was stored under −80°C until use.

### Library Construction and Metagenomic Sequencing

Libraries were constructed from 200 ng of isolated DNA using a Nextera XT DNA Sample Prep Kit (Illumina, USA) according to the manufacturer's protocol (24). The normalized libraries were diluted using hybridization buffer and then denatured by heat and spiked with 5% Illumina PhiX control DNA. Paired-end metagenomic sequencing was performed on an Illumina Novaseq-6000 platform by employing 2 × 150 base paired-end sequencing chemistry.

### Metagenomic Sequencing Data Processing and Analysis

Raw data files were converted to FASTQ files using Casava v.1.8.2 software. The quality of sequencing reads was controlled and contigs assembled using metaSPAdes v.3.10.1 (25). MetaGeneMark software (GeneMark.hmm v.3.38) was used to predict functional genes from assembled contigs (26). Putative amino acid sequences were aligned to proteins/domains using eggNOG (v.4.5) and the Kyoto Encyclopedia of Genes and Genomes (KEGG) databases (release 59.0) (27). Microbial community taxonomic profiles were determined by MetaPhlAn2 software (28). Taxonomic assignment of genes was performed using BLASTN and the NCBI-NT database. To identify antibiotic resistance genes in the gut microbiomes of piglets, amino acid sequences of corresponding genes were aligned against the Antibiotic Resistance Genes Database (ARDB) using BLASTP (*E*-value ≤ 1e-5) and considered antibiotic resistance Genesif the highest-scoring annotated hit had ≥80% similarity covering ≥70% of the query protein length (29). The Shannon index was calculated for each sample to determine α diversity. Bray–Curtis dissimilarity was utilized to compare differences in β diversity between different treatments using a permutational multivariate analysis of variance approach. Principal coordinate analysis based on Bray–Curtis dissimilarity distances was employed with visualization using R software.

### Statistical Analysis

All statistical analyses were carried out using *R* software. All comparisons were determined pairwise for every group. The Wilcoxon-test was used to analyze species and KEGG pathways, and *p* ≤ 0.05 was considered significant. All significance determination was at *p* ≤ 0.05.

## Results

### Effects of Colistin on Pig Gut Microbiome Composition

In this study, we used metagenomic sequencing analysis of the gut microbiomes of colistin-treated and control pigs to explore the influence of the antibiotic on microbiome composition. The analysis included 477,675,511 ± 7,084,183 clean reads from six pig stool samples, and 79,612,585 ± 7,084,183 sequences per stool sample were assessed (Table 1).

**Table 1:** Statistical summary of the pig gut microbiome.

**Samples**	**Raw reads**	**Clean reads**	**Clean ratio (%)**
Control	84,540,759 ± 3,995,921	81,769,114 ± 3,927,676	93.6 ± 0.2
Colistin	80,764,651 ± 10,542,548	77,456,056 ± 9,802,328	91.3 ± 1.4

### α-Diversity Analysis of Pig Gut Microbiome Composition

**Figure 1:**
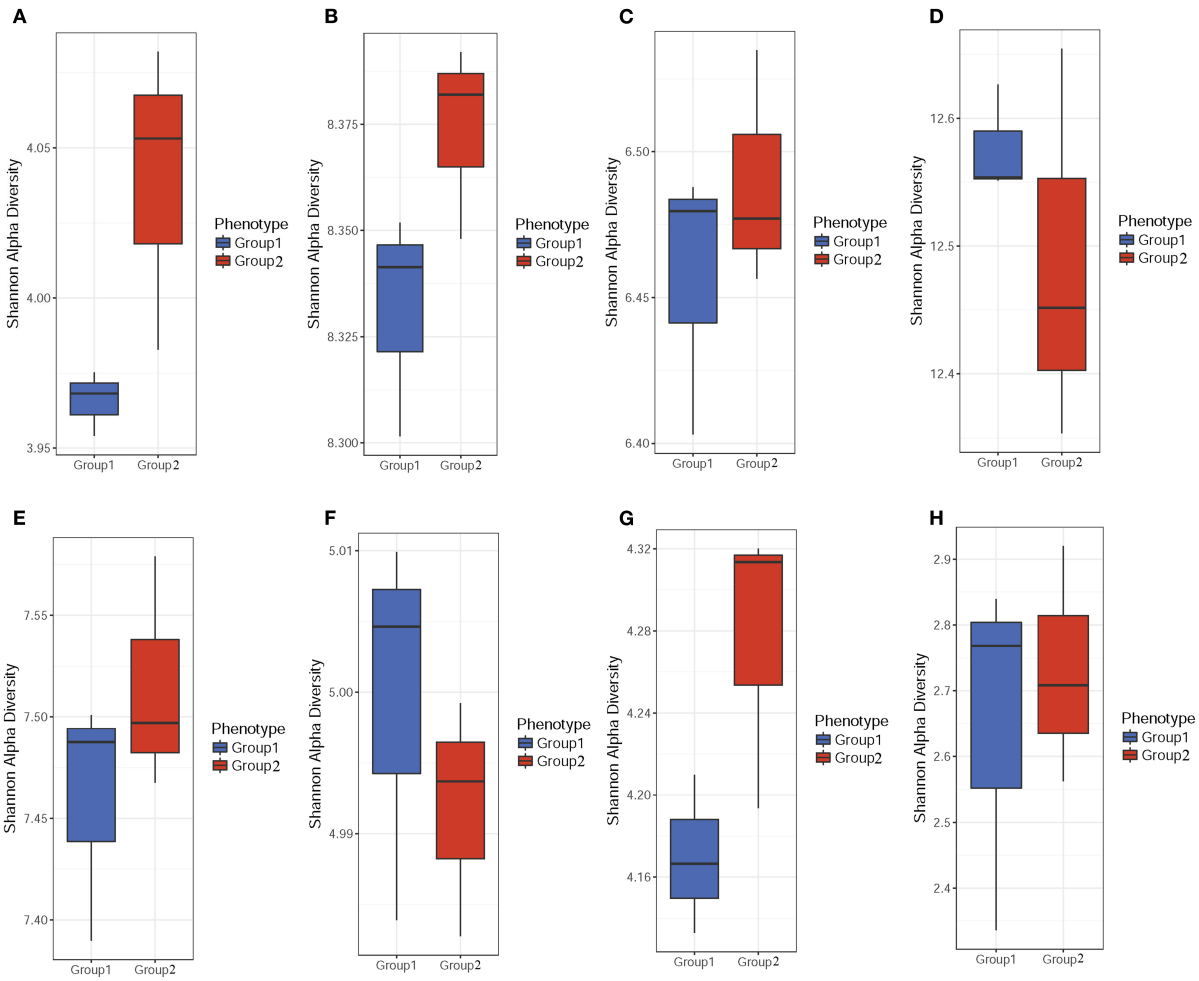
α-Diversity analysis of the pig gut microbiome composition. Stool samples were obtained from colistin and control groups. The Shannon *H* index was used to determine gut sample diversity using ARDB **(A)**, eggNOG **(B)**, Enzyme **(C)**, Gene **(D)**, KO **(E)**, Module **(F)**, Pathway **(G)**, and Taxonomy **(H)**.

Microbiome diversity in the community was determined using the Shannon diversity index (*H*) to measure individual sample richness and gut sample diversity. The results showed that samples from the colistin group had a more diverse microbiome community (Figures 1A–C,E,G,H), but control samples displayed higher abundance of gene and module than colistin samples based on the Shannon *H* index (Figures 1D,F). This might be due to the antibiotic colistin affecting gut microbial community diversity (17).

### β-Diversity Analysis of Pig Gut Microbiome Composition

**Figure 2:**
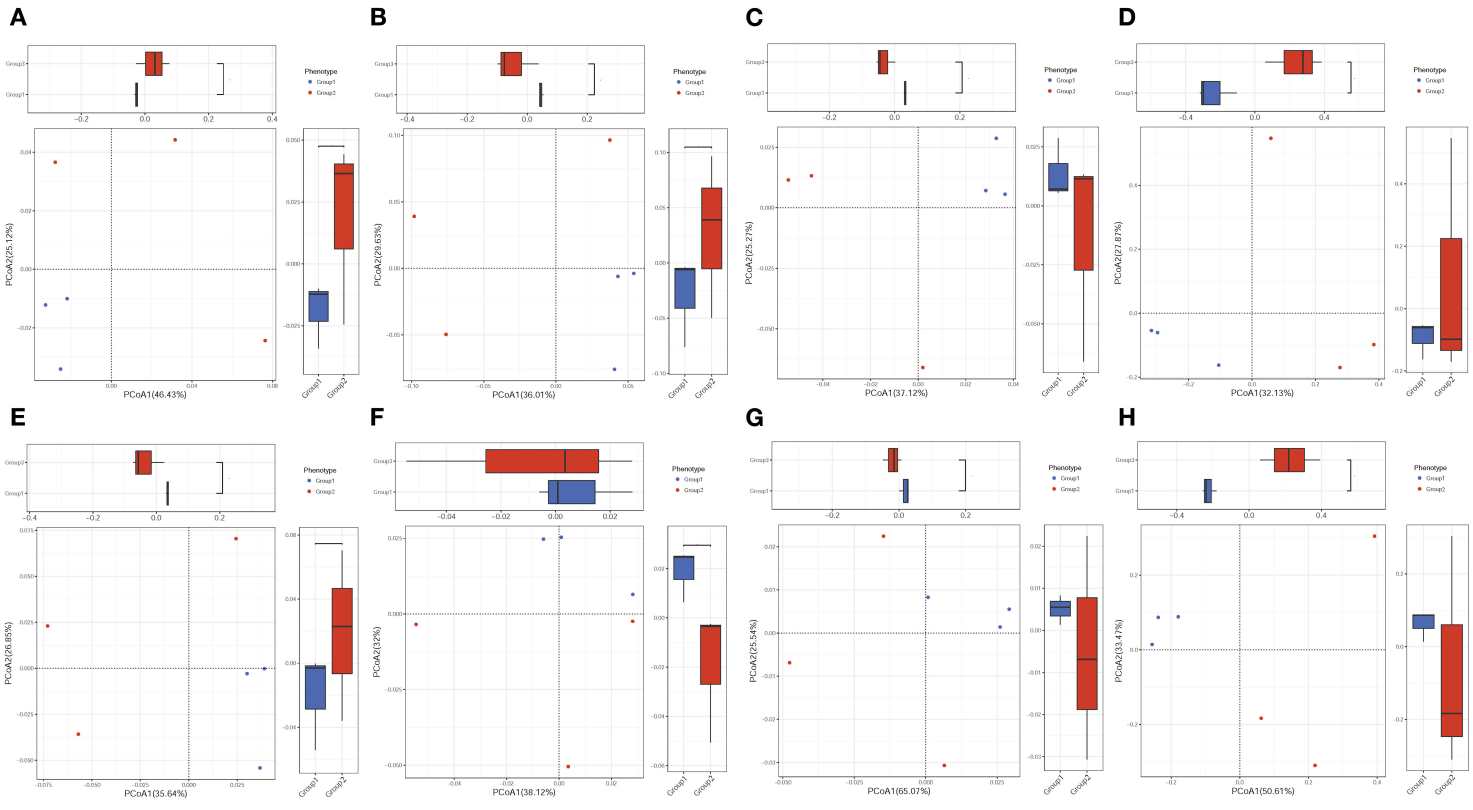
PCoA of the effects of colistin on the pig gut microbiome composition. PCA of gut microbiome changes for ARDB **(A)**, eggNOG **(B)**, Enzyme **(C)**, Gene **(D)**, KO **(E)**, Module **(F)**, Pathway **(G)**, and Taxonomy **(H)** for different groups. Group 1, control group; group 2, the colistin group.

PCoA analysis was performed to explore the associations between different samples. The results showed that most of the samples from both colistin and control groups were clustered together, but some were separate (Figure 2).

**Figure 3:**
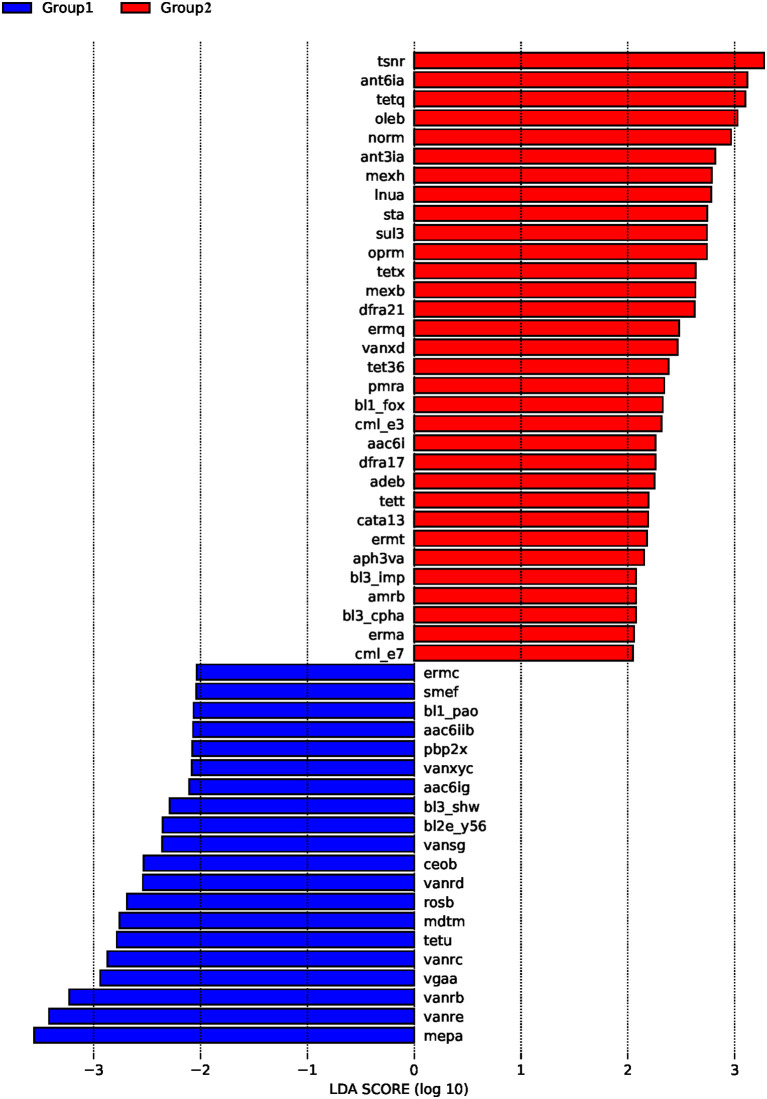
The effects of colistin on ARDB changes. Gut microbiome ARDB changes were analyzed by LEfSe. Group 1, control group; group 2, the colistin group.

ARDB changes were analyzed by LEfSe (Figure 3). The results demonstrated that in the control group, the main ARDB genes were *mepa, vanre, vanrb, vgaa, vanrc*, and *tetu* (Figure 3). However, following colistin treatment, expression levels of *tsnr, ant6ia, tetq, oleb, norm, ant3ia*, and *mexh* were significantly upregulated, indicating that colistin may induce transformation of antibiotic resistance genes (Figure 3).

### Effects of Colistin on Microbiome Composition at the Phylum Level

**Figure 4:**
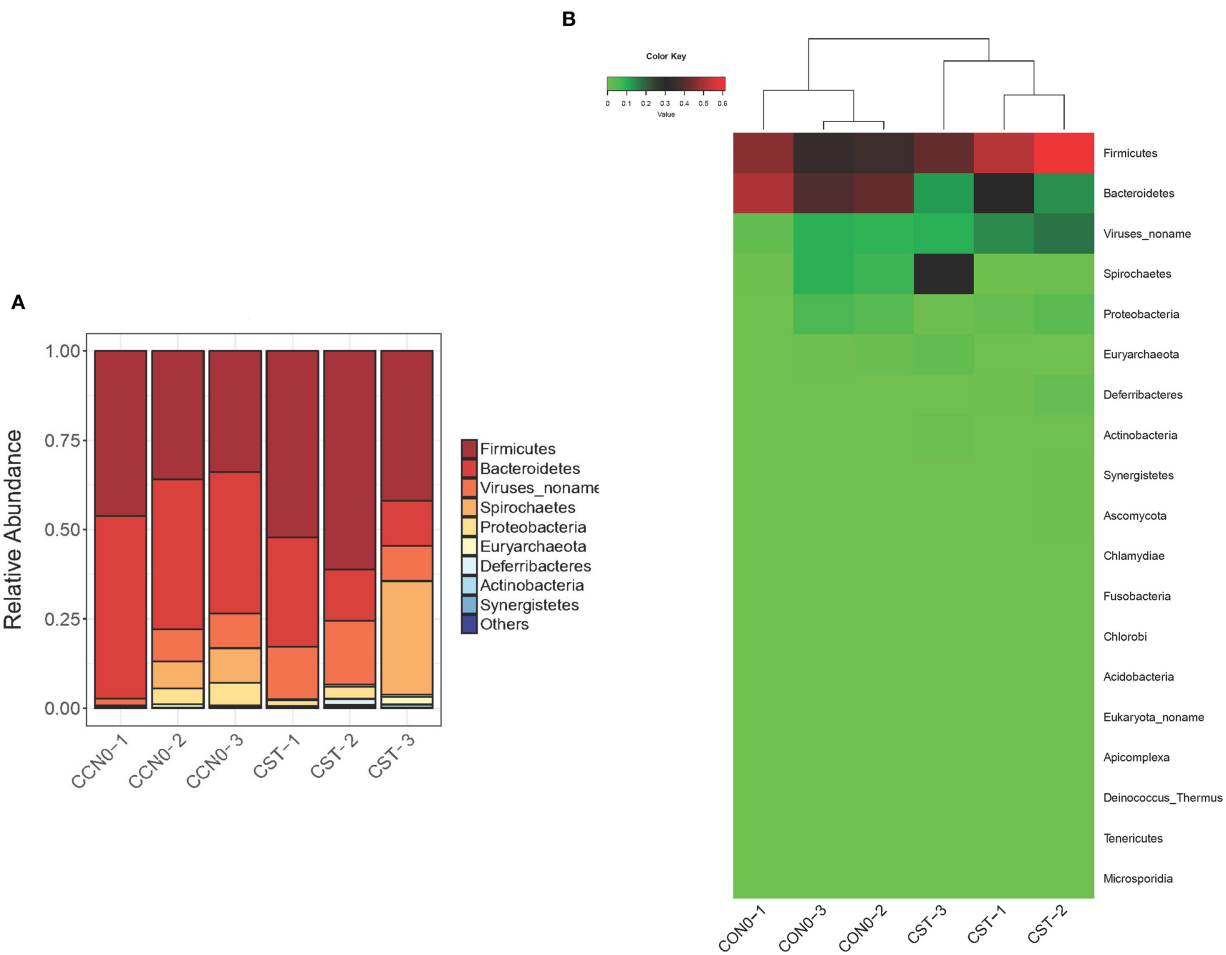
Effects of colistin on microbiome composition changes at the phylum level displayed as a bar chart **(A)** and a heatmap **(B)**. The abundance of phyla is shown on the *y* axis. Group 1, control group; group 2, colistin group.

We also analyzed the influence of colistin on microbiome composition at the phylum level, and the results demonstrated that colistin could alter the microbial profile (Figure 4). Firmicutes were the most abundant phylum in the colistin group, whereas Bacteroidetes were dominant in the control group (Figure 4). In addition, Spirochaetes were markedly abundant, whereas Proteobacteria and Euryarchaeota were significantly reduced when the pigs were administered colistin, compared with the control group (Figure 4).

### Effects of Colistin on Microbiome Composition at the Genus Level

**Figure 5:**
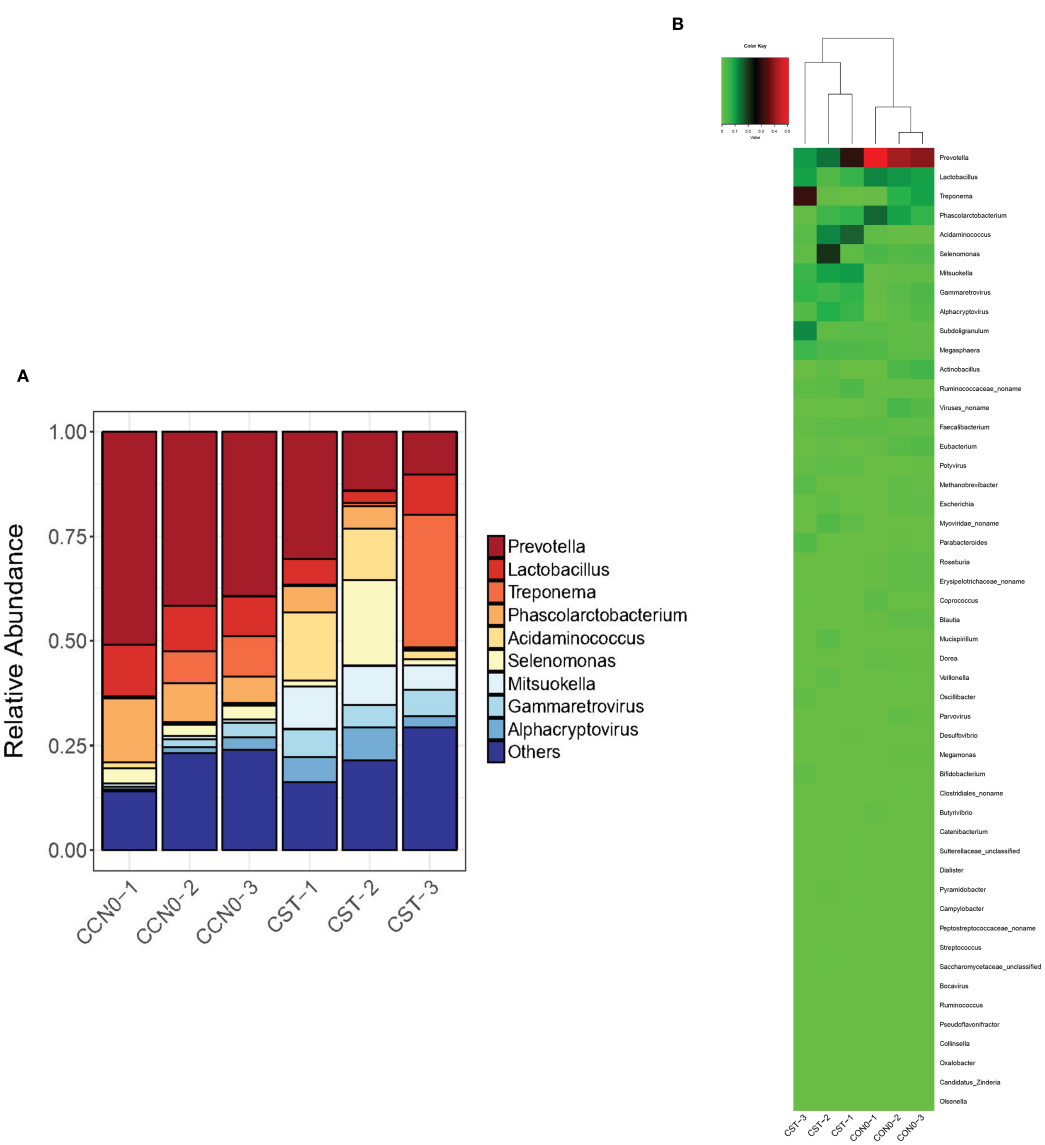
Effects of colistin on microbiome composition changes at the genus level displayed as a bar chart **(A)** and a heatmap **(B)**. The abundance of genera is shown on the *y* axis. Group 1, control group; group 2, colistin group.

Samples from control and colistin groups were assessed to evaluate the microbiome composition at the genus level. The results showed that, in general, colistin influenced the microbiome distribution pattern at the genus level (Figure 5). In the control group, the main components of the microbiome at the genus level were *Prevotella, Lactobacillus, Phascolarctobacterium*, and *Acidaminococcus* (Figure 5). However, following treatment with colistin, the abundance of *Prevotella, Lactobacillus, Phascolarctobacterium*, and *Acidaminococcus* was significantly decreased, whereas *Treponema, Selenomonas, Mitsuokella*, and *Gammaretrovirus* were significantly increased (Figure 5).

### Effects of Colistin on Microbiome Composition at the Species Level

**Figure 6:**
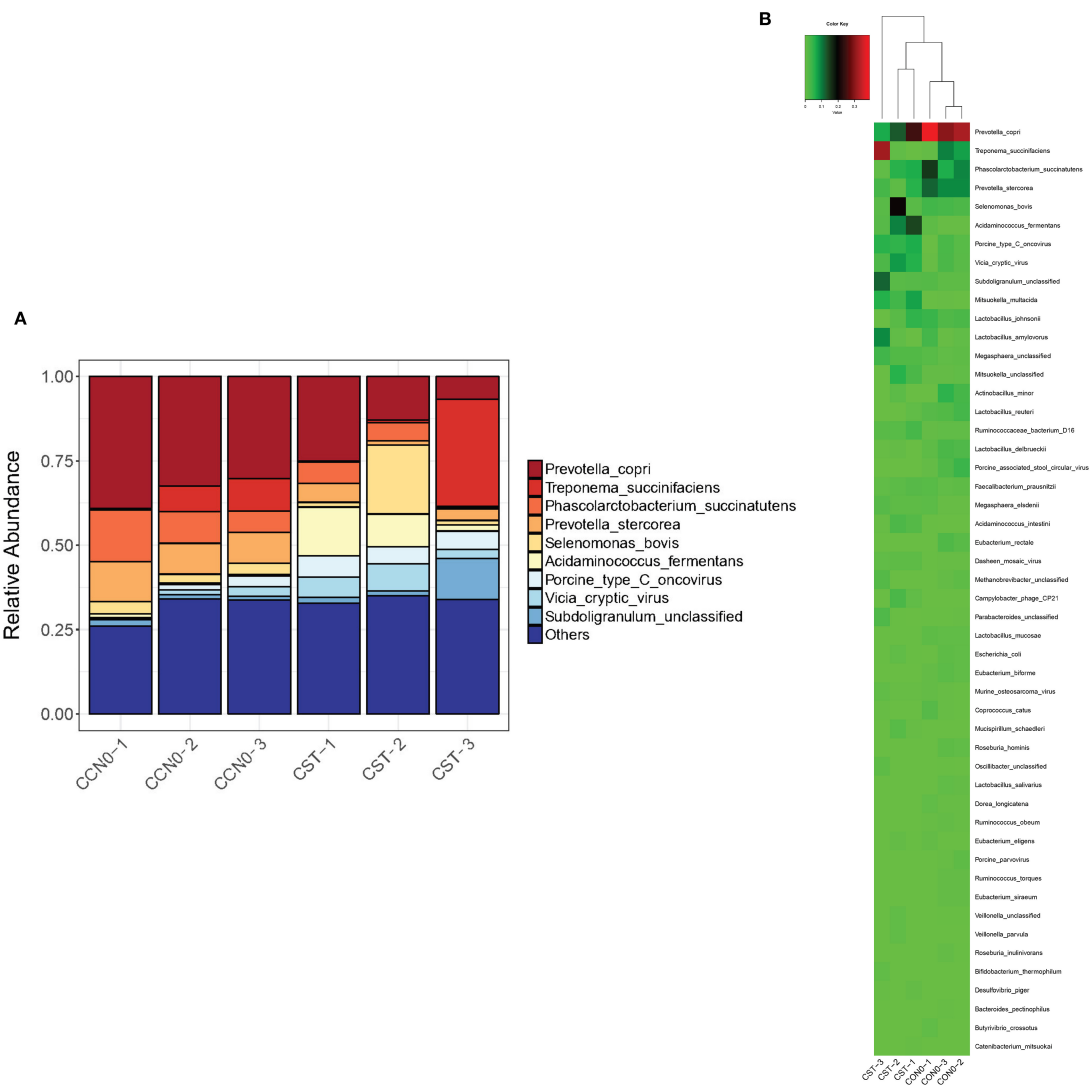
Effects of colistin on microbiome composition changes at the species level displayed as a bar chart **(A)** and a heatmap **(B)**. The abundance of species is shown on the *y* axis. Group 1, control group; group 2, colistin group.

Changes in microbiome composition at the species level were analyzed (Figure 6). The results showed that colistin significantly decreased the abundance of *Prevotella copri, Phascolarctobacterium succinatutens*, and *Prevotella stercorea* compared with the control group (Figure 6). The abundance of *Treponema succinifaciens* and *Acidaminococcus fermentans* was significantly increased in the colistin group (Figure 6).

### Effects of Colistin on Microbiome Composition Functional Changes

**Figure 7:**
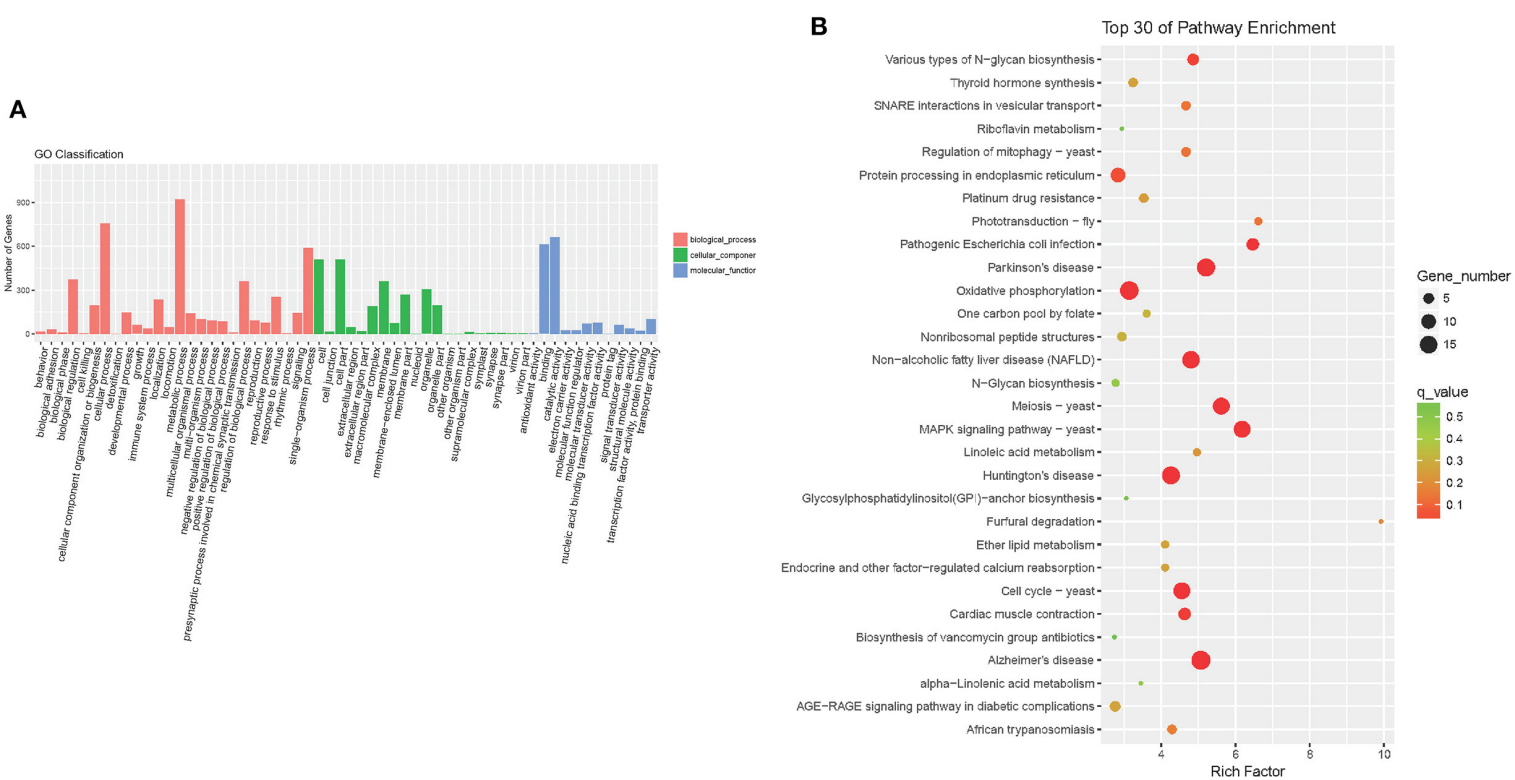
Detection of GO enrichment **(A)** and the top 30 signaling pathways identified by KEGG using DAVID analysis **(B)**.

To investigate the functions of differentially expressed genes, the Gene Ontology (GO) and KEGG analyses were performed. GO analysis showed that following treatment with colistin, metabolic process, cellular process, and single-organism process were the dominant terms in the biological process category, whereas binding and catalytic activity were the most enriched in the molecular function category (Figure 7A). KEGG analysis demonstrated that oxidative phosphorylation, protein processing in endoplasmic reticulum, various types of N-glycan biosynthesis, protein processing in endoplasmic reticulum, pathogenic *E. coli* infection, and mitogen-activated protein kinase (MAPK) signaling pathway–yeast were the dominant signaling pathways in the colistin group (Figure 7B).

## Discussion

This study was designed to compare the pig gut microbiome composition of colistin-treated and control animals. Colistin, produced by *Bacillus polymyxa*, has a strong antibacterial effect on Gram-negative bacteria (30) and is widely used on premixed feed to promote animal growth, but it is now forbidden in China as an additive to enhance animal growth (Announcement No. 2428 of the Ministry of Agriculture of the People's Republic of China). Colistin was recommended to treat extended-spectrum β-lactamase–producing Enterobacteriaceae (31). Colistin can inhibit *K. pneumoniae* biofilm formation and reduce interleukin 1β (IL-1β), tumor necrosis factor α, and IL-8 production (32). Nebulization of colistin is effective for the treatment of cystic fibrosis patients infected by *P. aeruginosa* (33). Although colistin can effectively enhance human and animal health, its application for this purpose is controversial because of the appearance of numerous colistin-resistant bacterial strains (34). Colistin has been reported to promote average daily gain, growth performance, and immunity responses in pig (35), but information on the relationship between colistin supplementation and the microbiome pattern in the pig gut is lacking. Our current study employed metagenomic sequencing analysis to explore the effects of colistin on microbiome diversity and changes in composition of the pig gut.

Gut microbiomes are considered a key factor in host physiology related to health and disease. The gut microbiome is considered as a possible key susceptibility factor for neurological disorders (36). The gut microbiome can also influence the human brain and behavior (37). Our results demonstrated that colistin treatment could increase piglet gut microbiome diversity. Previous research showed that land use affects microbiome diversity (38). Thus, disturbances in both a closed environment (gut) and an open environment (soil) can increase microbiome diversity. In the present study, at the species level, Gram-negative bacteria ghlighte *P. copri, P. succinatutens*, and *P. stercorea* were dominant in the control group, but all three were attenuated after pigs were fed colistin. Thus, colistin may exert a significant direct effect on microbiome composition and associated functions. It has been documented that *P. copri*, as the gut microbe, is immune-related and involved in rheumatoid arthritis pathogenesis (39), and the pathological processes of intestinal mucositis and gut toxicity are induced by carboplatin (40). In addition, *P. copri* is considered an ideal microbial marker for distinguishing high feed efficiency in beef cattle during life span and production cycles (41). *Prevotella copri* colonizes conventional mice and produces succinate to improve glucose homeostasis through intestinal gluconeogenesis (42). Thus, we speculate that *P. copri* might play important roles in maintaining gut function and health, but whether colistin altering the abundance of this gut microbe can affect gut flora balance and health requires further investigation.

Our current results showed that when piglets were treated with colistin, expression levels of *tsnr, ant6ia, tetq, oleb, norm, ant3ia*, and *mexh* were significantly upregulated, according to ARDB analysis. Expression of some potentially important genes, such as *mcr-1, mcr-9*, and *mcr-10*, was not been detected in the piglet gut following colistin treatment. Previous research reported that short-term treatment with colistin is correlated with the emergence of colistin-resistant Enterobacteriaceae in swine (43). The *mcr-1* gene was detected in colistin-resistant *S. enterica 4,[5],12:i:*-isolate from apparently healthy finishing pigs (44). Colistin-resistant *Salmonella typhimurium* harboring the *mcr-9* variant was found in Brazilian livestock (45). As these genes are at risk of being transmitted to humans through the food chain, we will study changes in antibiotic resistance genes following colistin treatment of piglets in more detail in the future.

We found that the abundance of *A. fermentans* was significantly upregulated following colistin treatment. We know relatively little about the function of this organism in maintaining gut health in pig. Previous research reported that this strictly anaerobic Gram-negative coccus may trigger autoimmunity responses by molecular mimicry, leading to ankylosing spondylitis (46), β-flavin adenine dinucleotide (FAD) produced by this bacterium bifurcates the electrons of NADH (47). The 2-hydroxyglutaryl-coenzyme A dehydratase enzyme of *A. fermentans* catalyzes reversible syn-elimination to (E)-glutaconylcoenzyme A (48). Thus, in future studies, we will isolate this bacterium to assess its impact on gut nutrition and health.

We also investigated the functions of gut bacteria genes through GO and KEGG analyses. Our findings revealed gene function differences between colistin and control groups. The functions of the dominant abundant genes in the colistin group were related to metabolic process, cellular process, binding, and catalytic activity. Some functions of differentially expressed genes were related to microbe community survival and proliferation, which was consistent with previous studies (49). However, one limitation to the present work was the relatively small number of pigs (three each in colistin and control groups) due to the high cost of pigs and metagenomic sequencing analysis. We also referred to the results of a previous study in which animals were used to explore microbiome composition changes (50). Interestingly, we found that the main signaling pathway (MAPK signaling pathway–yeast) was activated when pigs were fed colistin, according to KEGG analysis. This is the first report that the yeast MAPK signaling pathway in the pig gut can be activated. Previous research reported that the MAPK signaling pathway regulates the cell wall structure and ionic homeostasis in the fission yeast *Schizosaccharomyces pombe* (51), and stress-activated MAPK signaling inhibits the fission yeast *Schizosaccharomyces japonicus* from translocating to hypha (52). Inhibition of the MAPK signaling pathway can significantly attenuate *Saccharomyces cerevisiae* β-glucan–induced sheep β-defensin-1 expression (53). In addition, MAPK signaling controls cellular polarization triggered by pheromones in *S. cerevisiae* (54). Thus, we speculate that activation of the yeast MAPK signaling pathway might be related to environmental stress, such as antibiotic selection, which might be involved in the cell defense process, but the special mechanism needs further exploration.

## Conclusion

Taken together, our results demonstrated that colistin modified the composition and gene expression of the pig gut microbiome. Our findings might provide a new strategy for rational utilization of colistin in the clinic to maintain animal gut nutrition and public health.

## Data Availability Statement

The datasets presented in this study can be found in online repositories. The names of the repository/repositories and accession number(s) can be found at: NCBI SRA BioProject, Accession No: PRJNA720618.

## Ethics Statement

The animal study was reviewed and approved by Animal Care and Use Committee of Wuhan Polytechnic University, Hubei Province, China (EM950, November 5, 2020).

## Author Contributions

YQ conceived and designed the experiments. DZ, JZ, XZ, JH, CP, and YZ performed the experiments. LG, SF, CY, YL, ZW, and C-AH analyzed the data. LG, SF, and YQ wrote the paper. All authors contributed to the article and approved the submitted version.

## Conflict of Interest

The authors declare that the research was conducted in the absence of any commercial or financial relationships that could be construed as a potential conflict of interest.
